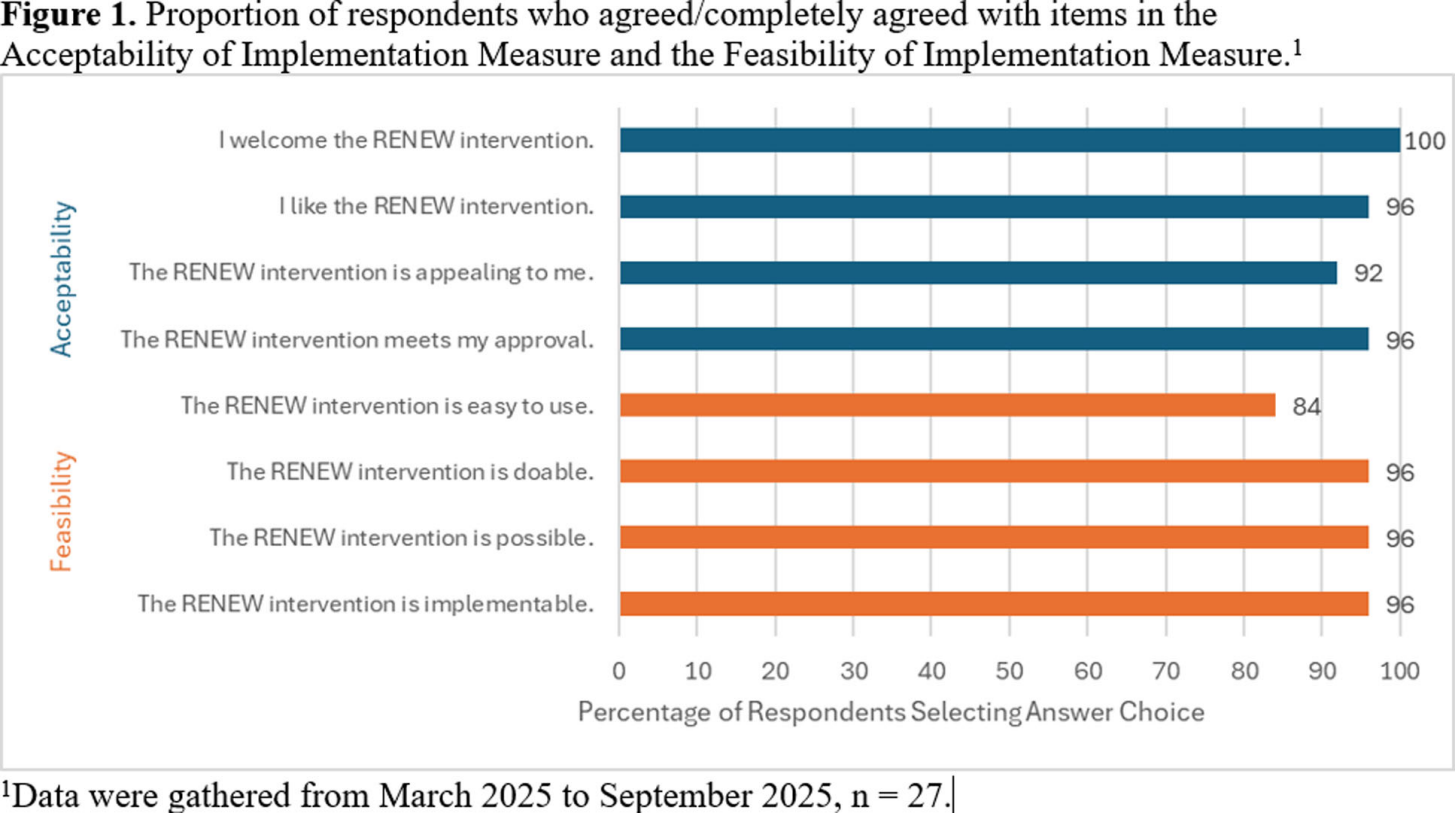# 78 Shared Surfaces, Shared Genomes: A Cross-Sectional Analysis of Multiple Multidrug-resistant Organisms in a Skilled Nursing Facility

**DOI:** 10.1017/ash.2026.10506

**Published:** 2026-06-23

**Authors:** Julie Szymczak, Brandi M. Muller, Anne Jaskowiak, Lauren Dutcher, Keith Hamilton, Robert Micheletti, Warren Bilker, Charles Leonard, Tyler Achuff, Rebecca Hirsh, Olajumoke Fadugba, Ebbing Lautenbach

**Affiliations:** 1 University of Utah School of Medicine; 2 University of Utah; 3 University of Pennsylvania Perelman School of Medicine; 4 University of Pennsylvania; 5 Hospital of the University of Pennsylvania; 6 Perelman School of Medicine, University of Pennsylvania

## Abstract

**Background:** Antibiotic allergy evaluation is an important antibiotic stewardship intervention, but is less commonly integrated into inpatient oncology care. Our objective was to evaluate the acceptability and feasibility of a pharmacist-driven penicillin allergy delabeling intervention for patients with hematologic malignancy (HM). **Methods:** We conducted a cross-sectional survey of physicians and advanced practice providers (APPs) who care for patients with HM at the Hospital of the University of Pennsylvania. The survey was administered from March 2025 to September 2025 in preparation for a planned Allergy Delabeling in Antibiotic Stewardship (RENEW) intervention. The survey included questions on current allergy evaluation practices, the perceived safety of RENEW and validated acceptability of intervention measure (AIM) and feasibility of intervention measure (FIM). AIM and FIM consist of 4 items scored 1-5 and is reported as a mean total score (5-20), with higher scores indicating greater acceptability and feasibility. Data were analyzed using descriptive statistics. **Result:** Of 70 eligible prescribers, 27 (38.6%) completed the survey: 21 (77.7%) physicians and 6 (22.2%) APPs. The majority (20, 74.1%) reported assessing documented antibiotic allergies of HM patients on admission and at the time an antibiotic is required (25, 92.6%). Nineteen (70.4%) respondents were comfortable modifying a documented antibiotic allergy in the medical record based on their assessment, while just under half (13, 48.1%) were comfortable deleting the allergy in the record based on their assessment. Nineteen (70.4%) respondents felt that penicillin skin testing was safe in patients with HM while 8 (29.6%) were not sure. Over half (14, 51.9%) were unsure if it is safe to evaluate penicillin allergy using an oral challenge based on clinical history without a skin test. The majority (22, 81.5%) said they would be comfortable having a patient’s antibiotic allergy removed from the medical record following negative allergy testing in the RENEW intervention. Compared to other patient safety initiatives, 24 (88.9%) believed evaluating documented antibiotic allergies is important or very important. Mean totals (SD; range) for acceptability and feasibility of the RENEW intervention were AIM, 17.2 (3.5; 8-20) and FIM, 17.7 (3.1; 8-20). **Conclusion:** Although the low response rate may limit generalizability, the majority of inpatient oncology clinicians perceived that a penicillin allergy delabeling intervention for patients with HM was acceptable and feasible to implement. Overall, clinicians believed that evaluating antibiotic allergy labels in patients with HM is an important and safe initiative, yet there was some uncertainty regarding the safety of a direct oral challenge.

**Figure:**